# Availability, use, and affordability of medicines in urban China under universal health coverage: an empirical study in Hangzhou and Baoji

**DOI:** 10.1186/s12913-018-2993-1

**Published:** 2018-03-27

**Authors:** Yunyu Huang, Youfen Jiang, Luying Zhang, Wenhui Mao, Job F. M. van Boven, Maarten J. Postma, Wen Chen

**Affiliations:** 1 School of Public Health, Fudan University, 130 Dong’an Road, Shanghai, 200032 China; 2 Unit of PharmacoTherapy, Epidemiology & Economics, Department of Pharmacy, University of Groningen, Antonius Deusinglaan 1, 9713 AV Groningen, The Netherlands; 3 Institute of Science in Healthy Aging & healthcaRE (SHARE), University Medical Center Groningen (UMCG), University of Groningen, Antonius Deusinglaan 1, 9713 AV Groningen, The Netherlands; 4 Department of Epidemiology, University Medical Center Groningen (UMCG), University of Groningen, Antonius Deusinglaan 1, 9713 AV Groningen, The Netherlands

**Keywords:** Medicines, Availability, Use, Affordability, Urban China, Universal health coverage

## Abstract

**Background:**

This study aimed to examine the availability, use, and affordability of medicines in urban China following the 2009 Health Care System Reform that included implementation of universal health coverage (UHC).

**Methods:**

This longitudinal study was performed in Hangzhou (high income, eastern China) and Baoji (lower income, western China). Five yearly household surveys were conducted (one each year from 2009 to 2013) to evaluate the impact of UHC on medicines use and expenditure, and a health facility survey was conducted in 2013 to evaluate availability of medicines. A cohort of over 800 households in Hangzhou and Baoji was established in 2009, and 20 hospitals were included in the health facility survey. Medicines use was determined using data from health facility and household surveys. An average, two-week out-of-pocket medicines expenditure was calculated to assess the affordability of medicines.

**Results:**

The number of medicines stocked in primary health facilities in Hangzhou decreased, while the number in Baoji increased. In Baoji, patients usually chose a pharmacy to buy medicines directly, despite the 48.2% increased availability of essential medicines in primary health care centers. The majority of survey respondents stated that their medicines need was basically met; however, medicines cost still accounted for a major part of their health expenditure. Medicines expenditure showed an increasing trend from 2009 to 2013. The average annual growth rate of household overall medical expenditure was significantly higher than that for household non-food consumption expenditure.

**Conclusions:**

Following China’s Health Care System Reform and implementation of UHC, availability and use of medicines has improved in urban areas. However, the affordability of medicines is still a concern.

**Electronic supplementary material:**

The online version of this article (10.1186/s12913-018-2993-1) contains supplementary material, which is available to authorized users.

## Background

In 2010, the World Health Organization (WHO) developed an operational guide for achieving universal health coverage (UHC) [1]. One of the goals of UHC is to ensure that individuals are able to afford health services and medicines. China is one of the low- and middle-income countries that has developed and implemented national strategies for UHC [2]. Following China’s Health Care System Reform in 2009 [3], UHC (comprising three main health insurance schemes) was preliminarily established to cover both urban and rural populations in China. The collective population covered by the rural New Cooperative Medical Scheme (NCMS), Urban Employee Basic Medical Insurance (UEBMI), and Urban Resident Basic Medical Insurance (URBMI) was > 98% of the total population of China in 2013 [4]. By expanding the UHC over the past decade, access to health care in China has been substantially improved [5].

Despite the broad population coverage, UHC in China is still in its primary stage [6]. Beneficiaries of these basic medical insurance schemes still have to pay a substantial proportion of health care expenditure as an out-of-pocket (OOP) expense [7, 8]. In 2010, expenditure on medicines in China accounted for > 40% of the total health expenditure [9]. One of the main reasons for this large percentage was the over-prescription and overuse of medicines (including antibiotics) in health facilities in order to generate greater revenues. Notably, this phenomenon mainly appeared after the marketization of the health care system during the implementation of the “reform and opening-up” program of economic reforms in 1978 in China [10, 11].

Noting that there were problems, the Chinese government began to develop appropriate policies and mechanisms through the implementation of health insurance schemes, the aim of which was to tackle overuse of medicines and control the rapid rise of health care costs — especially OOP payments [12, 13]. The health insurance system has acted as a third-party service purchaser, to ensure that service providers deliver high-quality services in an effective and efficient way.

The Health Care System Reform was a milestone in improving the health system in China. It was in 2009, under the reform, that the URBMI began to reimburse outpatient services (including medication) for the remaining population (i.e. those without coverage in the previous insurance system) in urban areas — largely comprising unemployed adults, the elderly, and children [14]. In addition, an essential drug policy was implemented that established a system of coverage for the main drugs needed by the public. These policy adjustments resulted in significant improvements in access to medicines, and reduced the financial burden placed on the service users — particularly those in low-income populations.

In recent decades, the availability, use, and affordability of (essential) medicines in China have been the subjects of extensive research [15–20], and the development of China’s essential medicines policies following the implementation of the Health Care System Reform in 2009 reviewed and analyzed in literature [21]. Findings of these studies/analyses showed that hospitals paid little attention to China’s National Essential Medicine List (NEML) in their decisions to purchase and stock essential medicines, and that there were worrying decreases in the availability of the lowest-price generic medicines in both the public hospitals and private pharmacies. Other studies have assessed medicine price, availability, and affordability using a methodology developed by the WHO and Health Action International [22–26]. The results of these studies showed that treating common diseases with low-price generics was generally affordable in China, whereas treatment with innovator brands was less affordable — especially for low-income populations. In addition, prescribing/use of essential medicines was frequently inappropriate. However, there is paucity of policy-oriented research on use of medicines in relation to design and implementation of specific national health insurance schemes across different socio-economic settings (e.g. high income eastern China versus lower income western China). One available study evaluated the impact of the Shenzhen labor health insurance scheme on access to essential medicines among migrant workers in 2007 [27]. However, the sample in this study was restricted to a local insurance scheme, which hampered extrapolation to national basic insurance coverage. Research on medicine use, availability, and affordability in relation to the national health insurance system following the 2009 reform could help to inform strategy and policy adjustments and improve affordable use of medicines under the current UHC in China.

This study aimed to examine the availability, use, and affordability of medicines in urban China after the implementation of China’s Health Care System Reform in 2009. In particular, the differences in medicine use and beneficiaries’ financial burden under the two major national urban basic medical insurance schemes (UEBMI and URBMI) were evaluated by comparing eastern China (high income, more health facilities) and western China (lower income, fewer health facilities).

## Methods

### Study design and setting

This study was a retrospective study that consisted of policy situational analysis, household surveys, and health facility survey in Hangzhou and Baoji.

Hangzhou (Zhejiang province) and Baoji (Shaanxi province) were selected as the sample cities based on their socio-economic level and health-system development, and as being representative of different regions in China. Both cities were included in the first batch of pilot cities implementing URBMI in 2009. These two cities were previously designated as Chinese national healthy cities, a national award to commend municipalities for their outstanding construction of health systems, which ensured that the surveys were efficiently conducted and the collected data were of good quality. In addition, the differences in socio-economic development (Table 1) and design of health insurance schemes in the two sample cities were apparent (Hangzhou: higher income, more health facilities; Baoji: lower income, fewer health facilities), which made these two cities typical urban representatives of eastern and western China that could be used to compare the regional UHC systems and outcomes of medicine supply and use.

**Table 1:** Basic socio-economic information of Hangzhou and Baoji in 2009

	Hangzhou	Baoji
Size of population^a^	8,100,000	3,731,400
GDP per capita^a^	63,471 CNY (9292 USD)	21,526 CNY (3151 USD)
Disposable income per capita^a^	26,864 CNY (3933 USD)	16,346 CNY (2393 USD)
Number of health institutions^b^	2687	903
Number of health workers per 1000 inhabitants^b^	8.23	4.13
Number of hospital beds per 1000 inhabitants^b^	5.89	4.23

^a^ Data source: Hangzhou / Baoji Economic and Social Development Statistics Bulletin 2009

^b^ Data source: Hangzhou / Baoji Health Development Statistics Bulletin 2009

*GDP* Gross Domestic Product

Definitions developed by the WHO were taken as reference to create the measures that allowed us to assess medicines availability, use, and affordability in China. Availability of medicines is defined by the WHO Health and Human Rights report, which states that medicines have to be available in sufficient quantity in public health and health care facilities [28]. The health facility survey was designed to determine the number of different medicines in stock in order to assess availability. Medicines use in this study was defined as the medicines taken or prescribed during patients’ health services, and patients’ perceptions of medicines use in daily life. Affordability of medicines was measured as the medicines expenditure and the ratio of medicines expenditure to total household resources [29]. The household surveys were conducted in the two sample cities, collecting residents’ data that allowed us to determine medicines use and affordability.

### Situational analysis

Relevant secondary data/information (including policy guidelines on insurance benefit packages, medicines policy and regulations, and local implementation plans related to UEBMI and URBMI in the two sample cities, etc.) were searched for and collected. Based on these materials, a situational analysis was performed to establish the basic background of the health insurance schemes and medicine policies in urban China, and in the two sample cities in particular. The content in the documents in written Chinese was translated by researchers from School of Public Health of the Fudan University in Shanghai, China (including the first and second authors of the present study). The translation referred to existing terms from literature [30–32] and was approved by reviewers from the funding organization (WHO).

### Household surveys

A cohort of over 800 households in Hangzhou and Baoji was established. A sample size of 2435 people was estimated using the hospitalization rate of 6.8% (obtained from the 4th National Health Services Survey in China) in order to ensure the power of statistical analyses for health-visit data. The sample size was calculated using the following formula [33]:

$$ \mathrm{n}={\mathrm{Z}}^2\mathrm{P}\left(1\hbox{-} \mathrm{P}\right)/{\updelta}^2 $$

Based on a degree of confidence of 95%, the statistic Z was set to 1.96. The P was set as the hospitalization rate of 6.8%, and the allowable error δ was set to 0.01. Based on the average number of three people per household, 800 households were designated to be investigated. Five household surveys were conducted (one each year from 2009 to 2013 in July/August to a total of five) to examine use of and expenditure related to health care services for urban residents. The survey used a structured questionnaire consisting of 7 sections: 1.) demographic information (e.g. age, sex, education, health insurance plan, etc.); 2.) perceived health needs (recent and chronic illness); 3.) service and medicines use during the past two weeks; 4.) costs of using health services during the past two weeks; 5.) patient satisfaction with services and medicines (including quality); 6.) views, knowledge, and attitudes on health insurance and medicines use; and 7.) household expenditure (monthly expenditure on food and non-food consumption including medical expenditure on chronic diseases, and annual expenditure on other specific matters in the last year, including medical expenditure on hospitalization or other specific doctor visits). The data in the first 6 sections were collected for each individual household member and the data in section 7 (household expenditure) for each household. The questionnaire design was based on the one used for the Chinese National Health Services Survey [33], which had been validated and improved during four previous national surveys.

#### Sampling

The household surveys used a multi-stage stratified random cluster sampling method. The urban districts were divided into 2 (Baoji) or 3 groups (Hangzhou) based on socio-economic level (good to poor). From each group, one district was selected. In each district, 3 (in Baoji) or 2 sub-districts (in Hangzhou) were randomly selected in order to pick 6 sub-districts in each city. Then, 3 communities (clusters) were sampled in each sub-district (overall 18 clusters in each city), and 45 households sampled in each community based on a random house-number allocation. Only family members with household registration (known as ‘Hukou’ in China) were investigated to ensure all included subjects were urban population. Due to the different response rate in different communities, the final number of households in each community was not exactly 45. However, the total number of households was guaranteed to exceed 800 in both cities.

#### Survey administration

An adequate number of investigators (> 20) selected from local universities in the study cities and Fudan University were trained by researchers from the Fudan School of Public Health (including first and second authors of this study) to conduct the surveys. The household surveys were conducted in five consecutive years (2009 to 2013) in order to continuously monitor services utilization and health care expenditure. Households could be censored due to moving, death, or ‘lost to follow-up’. In such situations, new families with household numbers close to that of the censored households were added to ensure the sample size of 800 households was maintained. Therefore, the number of households per year was not always exactly the same, but was maintained at more than 800 (Additional file 1: Table S1). The head of the household was the main respondent of the surveys. Parents answered questions relating to children younger than 16 years of age. The survey data were inputted by the investigators, and were checked by a different investigator in the research team.

### Health facility survey

In 2013, a health facility survey was conducted in Baoji and Hangzhou hospitals. In each city, 20 hospitals were identified. The proportion of primary, secondary, and tertiary hospitals was different in the two cities based on the local distribution of hospitals.

A structured questionnaire was designed to collect hospital information over three years from the two cities (2009, 2011, and 2012 for Baoji and 2009, 2011, and 2013 for Hangzhou). Key information on medicines use included general information such as hospital scale, revenue and spending, service amount delivered, and medicines purchasing and utilization.

### Data analysis

Availability, use, and affordability of medicines in the urban populations were assessed. Details are provided below.

#### Medicines availability

To evaluate availability, data from sampled health facilities was extracted from the health facility survey conducted in 2013. The number of different medicines stocked in the facilities (including number of different essential medicines, number of different antibiotics, and total number of different medicines included in the basic health insurance list) were analyzed to reflect the expected availability of medicines in urban health facilities.

#### Medicines use

General information on survey subjects’ health needs during the previous two weeks was extracted from the household survey (conducted from 2009 to 2013). Medicines use during the previous two weeks was analyzed in terms of percentage of patients taking medicines, the type of medicines taken (traditional Chinese medicine versus western medicine), and patients’ behavior and perception of taking medicines (choice of institutions for prescription, perception of dosage, and need). In addition, patients’ self-reported views and attitudes regarding medicines use were assessed. These view and attitude measures were only collected in the household survey conducted in 2013.

#### Medicines affordability

Due to the lack of explicit data on annual medicine expenditure, individual patient’s two-week OOP medicine expenditures (extracted from the household survey) were calculated to assess the economic burden of medicine expenditure for the years 2009 to 2013. Individual patient’s two-week expenditures for outpatient visits were also analyzed and compared with medicine expenditures. Patients’ two-week expenditures were collected in the household survey using the medical records kept by households, or self-reported information if there were no records to track. In addition, to explore household health-related economic burden, annual overall medical expenditure per household (calculated by summing up the annual chronic disease expenditure [monthly chronic disease expenditure multiplied by 12] and the annual specific medical expenditure) was compared with annual total household non-food consumption expenditure collected from the household surveys. Household income was not chosen as the comparator because of the unreliability of respondents’ self-reported income level [34]. All these expenditure measures were calculated for all 5 survey years in order to assess changing trends during the survey period. Expenditures were reported in 2009 US dollars using the exchange rate between US dollar (USD) and Chinese Yuan (CNY,) and adjusted for inflation by the Chinese National Consumer Price Index 2010 to 2013 [35].

#### Impact of socio-economic setting

All outcomes (medicines availability, use, and affordability) were calculated for Baoji and Hangzhou separately to assess the impact of socio-economic setting.

#### Statistics

Household survey data were entered into Epidata 3.1, and health facility survey data into Excel 2013. Descriptive and statistical analyses were performed using SPSS 18.0. Differences between UEBMI and URBMI / Baoji and Hangzhou were compared using several statistical tests. Expenditure data were reported using median, on account of the skewed distribution. Categorical data were compared using Chi-square tests. Continuous data were assessed using Mann-Whitney U-tests for comparisons between the two insurance schemes and cities, and using Kruskal-Wallis H-tests for comparisons between the five survey years (due to the non-normally distributed data [e.g. health expenditure]). Data from the health facility survey were not tested because of the low test power introduced by the small sample size.

## Results

### Situational analysis

Hangzhou’s UEBMI benefit package includes a pooling fund and a personal medical savings account. The pooling fund covers hospitalization reimbursement, and the personal account covers outpatient-visit reimbursement. Deductible, co-payment, and ceiling practices are employed as cost-sharing mechanisms to reduce patients’ moral hazard [36, 37]. All residents in the urban area and not covered by UEBMI are able to join URBMI on a voluntarily basis. URBMI does not have personal savings account, but has a similar pooling fund with a relatively higher co-payment by beneficiaries compared with UEBMI (approximately 50% co-payment percentage for URBMI versus 30% for UEBMI), in accordance with its lower required beneficiaries’ contribution. Our study indicates that there was a large gap between Hangzhou and Baoji regarding insurance benefit. For those insured by URBMI in Baoji, outpatient services were not covered, which led to a major burden for patients seeking regular medical services. In both cities, the differences between UEBMI and URBMI were only in the level of contribution to insurance financing and benefits for health services, while beneficiaries had the same accessibility to health services in all health institutions in different levels. A comparison between the two sample cities regarding the latest basic medical insurance plan designs is shown in Additional file 1: Table S2.

A national drug reimbursement list is issued to ensure people’s basic health care demands are met, and to eliminate unnecessary waste of resources through hierarchical reimbursement levels for listed services or medicines. The number of drugs on the 2010 Reimbursement Drug List is shown in Table 2. Class A drugs are those that are necessary for clinical use, and that demonstrate good effectiveness and a low price; these are reimbursed 100% by basic medical insurance. The list of Class A drugs is made by the central government, and is identical nationwide. Class B drugs are those that are optional for clinical use, and with similar effectiveness but higher price when compared with Class A drugs; these are partly reimbursed by basic medical insurance. The list of Class B drugs is made by the municipal government, and the reimbursement level differs based on municipal socio-economic level (Table 2). The drugs on the NEML are all included in Class A.

**Table 2:** Reimbursement drug list in Hangzhou and Baoji in 2010

	Class A^a^			Class B^b^		
	Western medicine	Chinese patent medicine	Reimbursement by insurance	Western medicine	Chinese patent medicine	Reimbursement by insurance
National-level	349	154	100%	791	833	–
Hangzhou	349	154	100%	866	951	80%, 97% and 99% based on drug types
Baoji	349	154	100%	983	961	85%

^a^ Class A drugs are those necessary for clinical use, with good effectiveness and low price. The list of Class A drugs is made by the central government and is identical nationwide

^b^ Class B drugs are those optional for clinical use, with similar effectiveness but higher price compared with Class A drugs. The list of Class B drugs is made by the municipal government

### Medicines availability

The general information on the service capacity of the investigated health facilities is shown in Additional file 1: Table S3. The service capacity of primary health care centers and tertiary hospitals in Hangzhou was greater than for those in Baoji, while the service capacity of secondary hospitals was lower in Hangzhou. Since implementation of the National Essential Medicine Policy in 2009, primary health care centers are required to stock and utilize medicines listed on the NEML — this list includes about 300 medicines plus local additions [38]. As a result, there was a sharp decrease in the number of different medicines (including antibiotics) stocked in primary health care centers in Hangzhou for the period 2009 to 2013 (Table 3), but the number of different medicines (including antibiotics) increased for the period 2009 to 2012 in Baoji. Notably, the number of different essential medicines in Baoji increased by 48.2% during this period (from 166 to 246). Since the NEML applies only to primary centers, the number of different medicines grew slowly in secondary and tertiary hospitals in both cities. However, a decreasing trend regarding number of antibiotics was found in secondary and tertiary hospitals in both cities.

**Table 3:** Numbers of different medicines stocked in health facilities

		Hangzhou			Baoji		
		2009	2011	2013	2009	2011	2012
Primary health care centers	Total number of different medicines	735	453	449	264	336	358
	Number of different medicines on NEML (percentage of total number)^a^	271 (37%)	235 (52%)	219 (49%)	166 (63%)	236 (70%)	246 (69%)
	Number of different antibiotics	49	19	18	19	24	26
Secondary hospitals	Total number of different medicines	813	832	849	635	643	638
	Number of different medicines on Insurance Reimbursement List	770	797	809	554	596	655
	Number of different antibiotics	59	53	38	39	36	35
Tertiary hospitals	Total number of different medicines	930	957	992	730	750	876
	Number of different medicines on Insurance Reimbursement List	808	841	913	677	716	805
	Number of different antibiotics	66	51	51	68	47	48

^a^ The National Essential Medicines Policy in China allows primary health care institutions to choose the medicines on the NEML and the additional provincial/municipal list. Although primary institutions can only equip essential medicines on the two lists, they are not mandatorily required to equip all medicines on the lists. So the numbers of NEML medicines were less than 307 in 2009 list, and rest of the stocked medicines were from the municipal list

*NEML* National Essential Medicines List

### Medicines use

In Hangzhou, UEBMI beneficiaries accounted for 60% of the household survey subjects; URBMI beneficiaries, less than 30%. In Baoji, the percentages of UEBMI and URBMI beneficiaries were both around 45% (Additional file 1: Table S4). In Hangzhou and Baoji, 30.7% and 23.9% (*p* < 0.001) of subjects, respectively, reported sickness in the two weeks before the 2013 survey (Table 4).

**Table 4:** Medicines use during past two-week period in 2013

2013	Hangzhou				Baoji				*p*-value of total population comparison between two cities
	Stratified by insurance scheme		*p*-value of comparison between two schemes	Total	Stratified by insurance scheme		*p*-value of comparison between two schemes	Total	
	UEBMI	URBMI			UEBMI	URBMI			
Prevalence during the past two weeks (among total subjects)	37.0% (695/1878)	17.3% (130/750)	< 0.001	30.7% (864/2885)^a^	30.1% (387/1287)	19.0% (230/1213)	< 0.001	23.9% (625/2615)^a^	< 0.001
Patients taking medicines	87.1%	86.5%	0.631	88.8%	91.7%	88.7%	0.078	89.9%	0.426
Type of medicines									
WM	8.4%	11.3%	0.020	9.1%	12.0%	14.7%	0.046	12.8%	< 0.001
TCM	67.6%	77.4%	< 0.001	69.2%	55.0%	58.6%	0.069	56.4%	< 0.001
Combination of WM & TCM	23.5%	11.3%	< 0.001	21.3%	32.4%	26.8%	0.002	30.5%	< 0.001
Not known	0.5%	–	–	0.4%	0.6%	–	–	0.4%	0.665
Type of institutions for medicines									
Primary station^b^	43.9%	39.8%	0.051	42.8%	8.2%	2.2%	< 0.001	6.0%	< 0.001
Primary center^b^	23.3%	25.0%	0.369	23.0%	7.2%	2.9%	< 0.001	5.5%	< 0.001
Secondary & tertiary	20.7%	16.7%	0.018	19.9%	4.6%	6.0%	0.109	5.2%	< 0.001
Private practice	0.3%	1.9%	< 0.001	1.1%	3.2%	3.8%	0.408	3.5%	< 0.001
Pharmacy	11.7%	16.7%	0.001	13.3%	74.8%	83.9%	< 0.001	78.1%	< 0.001
Knowing dosage	98.3%	97.2%	0.071	98.3%	98.9%	98.0%	0.101	98.6%	0.396
Following doctor’s instruction	96.4%	96.3%	0.892	96.1%	97.7%	97.5%	0.816	97.7%	0.001
Perception of whether medicines need was met									
Met	84.3%	91.2%	< 0.001	85.6%	96.3%	97.1%	0.366	96.6%	< 0.001
Partly met	12.0%	7.9%	0.002	11.4%	3.3%	2.6%	0.357	3.0%	< 0.001
Can’t be met	3.2%	0.9%	0.001	2.8%	0.2%	–	–	0.1%	< 0.001
Not known	0.5%	–	–	0.4%	0.2%	0.3%	0.648	0.2%	0.133

^a^ Total population included patients with all types of insurances, thus the number of patients in total population were larger than the sum of patient numbers from UEBMI and URBMI

^b^ Primary health care station: non-profit primary medical and public health service institution that serves for one community, affiliated to primary health care center; Primary health care center: non-profit primary medical and public health service institution that usually serves for one sub-district

*TCM* traditional Chinese medicine, *UEBMI* Urban employee basic medical insurance, *URBMI* Urban resident basic medical insurance, *WM* western medicine

Approximately 90% of patients in both cities took medicines during periods of sickness. Over half of those patients used traditional Chinese medicine, including both Chinese herbal medicine and Chinese patent medicine (Hangzhou: 69.2%; Baoji: 56.2% [*p* < 0.001]). Approximately 10% of patients used western medicine alone in the two sample cities (Table 4). Regarding choice of institution for obtaining medicines, there was a significant difference between the two sample cities. In Hangzhou, the majority of patients (65.8%) obtained medicines in primary health institutions; in Baoji, the majority (78.1%) obtained medicines in a pharmacy. In Hangzhou and Baoji, 98.3% and 98.6% of patients respectively, knew their medicine dosage (*p* = 0.396), and around 97% stated that they followed doctor’s instructions when taking medicines. There were more respondents stating that their medicines needs could be met in Baoji (96.6%) than in Hangzhou (85.6%) (*p* < 0.001).

In both cities, the self-reported prevalence was higher in UEBMI beneficiaries than in URBMI beneficiaries (*p* < 0.001 in both cities). More URBMI beneficiaries than UEBMI beneficiaries chose a pharmacy to fill a prescription (*p* = 0.001 in Hangzhou; *p* < 0.001 in Baoji). In Hangzhou, more URBMI beneficiaries than UEBMI beneficiaries stated that their medicines needs had been met (*p* = 0.035). Different insurance schemes did not influence patient-reported dosage and prescription compliance.

### Medicines affordability

**Fig. 1:**
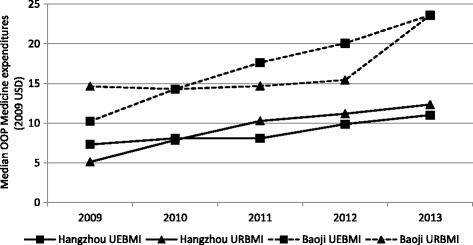
Comparison of OOP medicine expenditures in the two weeks before each survey (median). Abbreviations: OOP, out-of-pocket; UEBMI: Urban employee basic medical insurance; URBMI: Urban resident basic medical insurance

In general, median OOP medicine expenditure in the two weeks before each survey was higher in Baoji than in Hangzhou (Fig. 1). In both cities, and for both insurance schemes, the medicine expenditure showed an increasing trend from 2009 to 2013. The results did not suggest that medicine expenditures were different between the insurance schemes (9 out of 10 *p*-values for comparison between two schemes were larger than 0.05; Additional file 1: Table S5). Compared with the average expenditure on outpatient visits in the same past two weeks, OOP medicine expenditure accounted for 26–51% of outpatient expenditure in Hangzhou and 30 to 51% in Baoji.

There was a significant increasing trend in household overall medical expenditures in both cities for the period 2009 to 2013 (*p* < 0.001 for the 5 years of comparison for both cities; expenditures were significantly not all similar for the 5 years). The median annual household overall medical expenditure was higher in Hangzhou, and increased from 358 USD to 578 USD from 2009 to 2013, while it increased from 249 USD to 415 USD in Baoji during the same time period. The average annual growth rate of medical expenditure in 5 years (12.8% in Hangzhou; 13.6% in Baoji) was much higher than the growth rate of household non-food consumption expenditure (1.3% in Hangzhou; 5.0% in Baoji).

## Discussion

This study analyzed medicines availability, use, and affordability in urban China following the implementation of the Health Care System Reform 2009 and UHC. To our knowledge, there are no empirical studies using longitudinal data to assess medicines use following UHC implementation in China. Our results show that the availability of medicines in hospitals, the use of medicines, and the affordability of medicines varied between the two sample cities and health insurance schemes for the period of study. Different types of urban basic health insurance (i.e. UEBMI and URBMI) influenced patients’ behavior when seeking health services and medicines (e.g. choice of institutions for obtaining medicines), but did not show clear impact on medicine expenditures. The results also show that patients’ OOP medicine expenditures increased significantly over the period 2009 to 2013, and that household overall medical expenditure increased much greater than non-food consumption expenditure.

### Medicines availability

After implementation of UHC in China, the availability of medicines in health facilities was improved. A previous study showed greater availability of low-priced generics in primary health care facilities [26]. The present study demonstrated improved availability of essential drugs and improved drug stock in primary health facilities for the study period. The National Essential Medicine Policy requires primary health facilities to stock and prescribe essential medicines, and regulates medicine bidding and purchasing strictly. Our results showed that the proportion of essential medicines increased from 37% in 2009 to 49% in 2013. After the establishment of UHC, the government accelerated the development of primary institutions and increased their service capacities. However, the socio-economic development level was still a major determinant for medicine availability. Health care facilities in cities with higher socio-economic level, (e.g. Hangzhou) were allocated more medicines overall.

### Medicines use

Under the national guidance for developing UHC, local health insurance authorities were encouraged to implement municipality-wide health insurance schemes tailored to local socio-economic situations. This decentralization arrangement has led to great differences in health insurance schemes, benefit packages, and patients’ health-related financial burden [39]. Even within the same national Chinese health insurance schemes, there were major differences in local implementation regarding service coverage for outpatient and inpatient care and availability of medicines, mainly due to different levels of socio-economic development. In Hangzhou (high socio-economic level), URBMI covered outpatient visits — this was not the case in Baoji. This difference in insurance schemes might have influenced patients’ medicines use: for example, UEBMI beneficiaries were more likely to take medicines than URBMI beneficiaries due to the higher level of health insurance benefits. These results are consistent with previous evidence that indicates that differences in local UEBMI and URBMI schemes may increase inequities in the use of outpatient services — for example, that high-income populations utilize outpatient services more than low-income populations [40]. However, URBMI has positively increased the use of inpatient services [41, 42]. In general, and despite the potential inequity introduced by different health insurance schemes, our results showed that urban population were mostly satisfied with their medicines use based on their self-reported perception.

### Medicines affordability

Although studies have shown that encouraging the use of essential medicines is effective in curtailing prescription medicines costs [20], the affordability of medicines is still a challenge in China [25]. Medicines expenditure increased significantly during the 5-year study period, and the cost of medicines accounted for a major part of outpatient-visit expenditure. Our health facility survey showed that medicines revenue, as a proportion of total revenue, for health institutions was 50% in Hangzhou and > 30% in Baoji. The percentage contribution of household overall medical expenditure to total non-food consumption expenditure was significantly higher in the sample cities than in high-income countries (12.8% in Hangzhou and 13.6% in Baoji versus around 8% in U.S.) [43]. The reported growth rate of annual household medical expenditure was much higher than annual household consumption expenditure, which reflected that the growing trend of increasing medical costs was still a financial threat to Chinese urban residents — especially those at a lower socio-economic level. The health insurance schemes didn’t seem to influence medicine affordability; there were no significant disparities between medicine expenditure in UEBMI and URBMI schemes. The Chinese government still has a long way to go when it comes to reducing the medical-economic burden and changing the perception that seeing a doctor is expensive.

### Limitations

The main limitation of this study was the difficulty in inferring causality between the implementation of UHC and differences in medicines availability, use, and affordability. The UHC scheme, and the supporting policies that were implemented following the Health Care System Reform in 2009, were rapidly developed and subsequently adjusted. Thus, changes in the availability, use, and affordability of medicines may not be directly explained by any one single policy. Monitoring practices, and a continuous analysis of China’s health system, need to be promoted to encourage further research and assessment. Large scale electronic databases, including administrative or insurance claims databases, could be developed — ideally quickly, preferably to a standard protocol — to provide continuous evidence gathered from across the country. Other limitations of this study were mainly related to the data collection in the household surveys. Although randomization was conducted as strictly as was possible when sampling communities, there were still factors that influenced selection bias in the surveys. The inclusion of households was dependent on respondents’ willingness to participate in the surveys. The elderly people, and highly-educated people, were more cooperative. The difference in data from included subjects and those who refused to participate in surveys was difficult to evaluate. Recall bias might also influence the accuracy of the self-reported medicine-use data and the income and expenditure data. For example, those who were familiar with their own medical condition and used medicines regularly might report more accurate medicine-use data, and respondents’ perceptions of privacy and money might influence the accuracy of income and expenditure data.

## Conclusions

In conclusion, after China’s Health Care System Reform in 2009 and the accompanying implementation of UHC, the availability and use of medicines has improved — but affordability of medicines is still a concern for policy makers. Improvements in policies focusing on narrowing the gap between health insurance schemes and regulating medicine allocation and use are highly recommended.

## Additional file

Additional file 1: **Table S1** The number of households and subjects surveyed between 2009 and 2013. Table shows the number of households and subjects surveyed in the five yearly household surveys. **Table S2** Comparison of health insurance schemes in Hangzhou and Baoji in 2013. Table shows the comparison between the two sample cities regarding the latest basic medical insurance plan designs. **Table S3** General Information about the size of the Health Facilities. Table shows the general information on the service capacity of the investigated health facilities in Hangzhou (2013) and Baoji (2012). **Table S4** General information of UEBMI and URBMI beneficiaries. Table shows the general information of UEBMI and URBMI beneficiaries in the two sample cities in the five yearly household surveys. **Table S5** Comparisons of out-of-pocket medicine expenditures in the two weeks before each survey. Table shows the comparisons of the out-of-pocket medicine expenditures between the two sample cities in the two weeks before each survey. (DOCX 24 kb)

## Abbreviations

NCMS: New cooperative medical scheme
NEML: National Essential Medicine List
OOP: Out-of-pocket
UEBMI: Urban employee basic medical insurance
UHC: Universal health coverage
URBMI: Urban resident basic medical insurance
WHO: World Health Organization
