# Supplementary material for: Availability, use, and affordability of medicines in urban China under universal health coverage: an empirical study in Hangzhou and Baoji
Source: BMC Health Serv Res. 2018 Mar 27;18:218. doi: 10.1186/s12913-018-2993-1 (PMC5870255; doi:10.1186/s12913-018-2993-1)
Supplement: Supplementary file 1 — Table S1 The number of households and subjects surveyed between 2009 and 2013. Table shows the number of households and subjects surveyed in the five yearly household surveys. Table S2 Comparison of health insurance schemes in Hangzhou and Baoji in 2013. Table shows the comparison between the two sample cities regarding the latest basic medical insurance plan designs. Table S3 General Information about the size of the Health Facilities. Table shows the general information on the service capacity of the investigated health facilities in Hangzhou (2013) and Baoji (2012). Table S4 General information of UEBMI and URBMI beneficiaries. Table shows the general information of UEBMI and URBMI beneficiaries in the two sample cities in the five yearly household surveys. Table S5 Comparisons of out-of-pocket medicine expenditures in the two weeks before each survey. Table shows the comparisons of the out-of-pocket medicine expenditures between the two sample cities in the two weeks before each survey. (DOCX 24 kb) [file 12913_2018_2993_MOESM1_ESM.docx]

**Table S1 The number of households and subjects surveyed between 2009 and 2013**

|  | **2009** | **2010** | **2011** | **2012** | **2013** |
| --- | --- | --- | --- | --- | --- |
| **Number of households** |  |  |  |  |  |
| Hangzhou | 838 | 819 | 843 | 863 | 830 |
| Baoji | 867 | 878 | 919 | 919 | 877 |
| **Number of subjects** |  |  |  |  |  |
| Hangzhou | 2717 | 2755 | 2749 | 2892 | 2885 |
| Baoji | 2609 | 2567 | 2802 | 2715 | 2615 |

**Table S2 Comparison of health insurance schemes in Hangzhou and Baoji in 2013^*^**

| **Hangzhou** | | **Baoji** | |
| --- | --- | --- | --- |
| **UEBMI** | **URBMI** | **UEBMI** | **URBMI** |
| **Contribution:** | | | |
| - Employer: 6–11.5% salary  - Employees: 2% salary  - Flexible employees: 5–9% average wage of last year in Zhejiang | - Children: 400 (government subsidy 250)  - College students: 120 (government subsidy 90)  - Elderly and adults:  a) Package A: 1200 (government subsidy 800)  b) Package B: 800 (government subsidy 600) | - Employer: 6% salary  - Employees: 2% salary  - Flexible employees: 8% average wage of last year in Baoji | - Children: 320 (government subsidy: 300)  - College students: 320 (government subsidy: 300)  - Elderly and Adults: 450 (government subsidy: 300) |
| **Benefits for outpatients:** | | | |
| Deduction:  - Personal medical saving account of current year reimburses outpatient visits first;  - Deduction after personal account is consumed: 1000 for in-service employees, 700 for retirees | Deduction:  300 per year | Deduction:  Personal medical saving account of current year reimburses outpatient visits;  No reimbursement for common diseases after personal account is consumed. | Deduction:  No reimbursement for outpatient visits (except college students) |
| Co-payment:  Decided by government of the pooling region, ≥ 70% | Co-payment:  - Children: 40%, 50% and 60%**  - Elderly and Adults:  a) Package A: 40%, 50% and 70%**;  b) Package B: 25%, 35% and 60%** | Co-payment:  Risk pooling of chronic diseases | Co-payment:  Risk pooling among college students |
| Ceiling:  Decided by pooling region | Ceiling:  No ceiling. | - | - |
| **Benefits for inpatients:** | | | |
| Deduction:  800, 600, and 300** per year | | Deduction:  600, 400, 150** per year | Deduction:  - Children & college students: 300, 200,100**  - Adults: 600, 400,150** |
| Co-payment:  Deduction to ceiling: decided by government of the pooling region, ≥80% | Co-payment:  - Children and college students:  a) Deduction to 20000: 64%, 70% and 76%**  b) 20000 to 40000: 70%, 75% and 80%**  c) 40000 to 150000: 76%, 80% and 84%**  d) >150000: 82%, 85%, and 88%**(only for college students)  - Elderly and adults: Deduction to ceiling: 65%, 70% and 75%* * | Co-payment:  - In-service employees:  Deduction to ceiling: 88%, 90%, 92%**  - Retirees:  Deduction to ceiling: 90%, 92%, 94%** | Co-payment:  - Adults:  Deductible to ceiling: 60%, 70%, 85%** (90% for 2011, 2012 and 2013)  - Children and college students:  a) deduction to 5000: 50%, 60%, 80%**  b) 5000 to 10000: 55%, 70%, 85%**  c) 10000 to 24000: 60%, 80%, 90%**  d) 24000 to 70000: 80%, 85%, 95%**  e) >70000: 85%, 90%, 95%** |
| Ceiling:  ≥ 6 times of AAW | Ceiling:  ≥6 times of API | Ceiling  130000 | Ceiling:  130000 for children & college students and 90,000 for adults |
| Medical cost above the ceiling is shared by individual and Critical illness Subsidy Fund | | Medical cost above ceiling and below next higher ceiling is shared by individual and Critical illness Subsidy Fund | |
|  |  | Higher ceiling:  130000 to 200000: 90% for in-service employees and 92% for retirees | Higher ceiling:  a) Lower ceiling to 130000: 85%;  b) 130000 to 300000: 90% |
| **Insurance payment method to health facilities:** | | | |
| Global budget for each facility | | Fee for service | |

AAW: Annual average wages of staff and workers; API: average per capital disposable income of rural and urban residents; UEBMI: Urban employee basic medical insurance; URBMI: Urban resident basic medical insurance.

* Unit of the amounts in the table is CNY.

** For tertiary hospital, secondary hospital and primary hospital accordingly.

**Table S3 General Information about the size of the Health Facilities**

|  | **Hangzhou (2013)** | | | **Baoji (2012)** | | |
| --- | --- | --- | --- | --- | --- | --- |
|  | **Primary** | **Secondary** | **Tertiary** | **Primary** | **Secondary** | **Tertiary** |
| Number of investigated facilities | 6 | 2 | 12 | 5 | 5 | 10 |
| Area (m^2^) | 5064 | 23130 | 69287 | 3031 | 28092 | 48250 |
| Number of staff | 134 | 426 | 1362 | 56 | 501 | 1180 |
| Number of practitioners | 54 | 132 | 406 | 18 | 127 | 291 |
| Number of nurses | 36 | 159 | 577 | 18 | 187 | 458 |
| Number of beds | 38.9 | 340.0 | 1060.6 | 49.7 | 298.3 | 897.2 |
| Number of outpatient and emergency visits/year (thousands) | 684.7 | 252.7 | 1358.0 | 37.6 | 123.0 | 389.5 |
| Number of outpatient prescriptions/year (thousands) | 306.3 | 396.7 | 1760.7 | 22.1 | 82.9 | 312.8 |
| Number of patients discharged/year | 188.3 | 8676.5 | 29973.4 | 1114.1 | 9724.0 | 30382.0 |

**Table S4 General information of UEBMI and URBMI beneficiaries**

|  | **2009** | | **2010** | | **2011** | | **2012** | | **2013** | |
| --- | --- | --- | --- | --- | --- | --- | --- | --- | --- | --- |
|  | **UEBMI** | **URBMI** | **UEBMI** | **URBMI** | **UEBMI** | **URBMI** | **UEBMI** | **URBMI** | **UEBMI** | **URBMI** |
| **Hangzhou** |  |  |  |  |  |  |  |  |  |  |
| Gender: Male | 50.2% | 44.4% | 49.8% | 46.0% | 49.5% | 45.9% | 49.1% | 48.2% | 48.9% | 47.2% |
| Female | 49.8% | 55.6% | 50.2% | 54.0% | 50.5% | 54.1% | 50.9% | 51.8% | 51.1% | 52.8% |
| Age (years, mean±SD [25^th^, 75^th^ percentile]) | 51.7±16.5  (38,64) | 34.5±25.0  (10,58) | 52.8±16.8  (38,66) | 33.8±24.7  (10,55) | 53.6±17.5  (38,68) | 34.3±25.0  (11,58) | 54.2±17.5  (39,67) | 34.9±24.8  (12,56) | 54.4±17.4  (38,68) | 30.7±24.8  (9,53) |
| Beneficiaries’ percentage in total population* | 60.6% | 29.7% | 63.2% | 27.8% | 61.6% | 28.6% | 65.4% | 27.1% | 65.1% | 26.0% |
| **Baoji** |  |  |  |  |  |  |  |  |  |  |
| Gender: Male | 55.4% | 39.4% | 57.0% | 39.2% | 55.5% | 41.5% | 57.0% | 39.5% | 55.7% | 42.7% |
| Female | 44.6% | 60.6% | 43.0% | 60.8% | 44.5% | 58.5% | 43.0% | 60.5% | 44.3% | 57.3% |
| Age (years, mean±SD [25^th^, 75^th^ percentile]) | 51.5±14.5  (40,62) | 34.9±21.6  (16,50) | 53.1±15.4  (40,61) | 36.0±22.3  (12,50) | 52.9±14.7  (41,63) | 34.6±20.8  (17,48) | 54.2±15.5  (42,66) | 37.5±22.0  (18,50) | 54.9±15.6  (42,67) | 36.7±21.7  (18,50) |
| Beneficiaries’ percentage of total population* | 45.8% | 41.4% | 48.8% | 42.3% | 47.9% | 45.4% | 46.9% | 47.1% | 49.2% | 46.4% |

* Percentages do not sum up to 100% as the remaining part had rural or no insurance.

UEBMI: Urban employee basic medical insurance; URBMI: Urban resident basic medical insurance.

**Table S5 Comparisons of out-of-pocket medicine expenditures in the two weeks before each survey**

|  |  | **2009** | **2010** | **2011** | **2012** | **2013** | **Comparison between 5 years (p-value)** |
| --- | --- | --- | --- | --- | --- | --- | --- |
| Comparison between two schemes (p-value) | Hangzhou | 0.400 | 0.020 | 0.126 | 0.546 | 0.967 | UEBMI: 0.045  URBMI: 0.018 |
|  | Baoji | 0.480 | 0.205 | 0.044 | 0.400 | 0.434 | UEBMI: <0.001  URBMI: 0.041 |
|  |  |  |  |  |  |  |  |
| Comparison between two cities (p-value) | UEBMI | 0.002 | 0.020 | <0.001 | <0.001 | <0.001 |  |
|  | URBMI | 0.715 | 0.831 | <0.001 | 0.303 | <0.001 |  |
|  |  |  |  |  |  |  |  |
| Medicine OOP expenditures as percentage of outpatient expenditure during the past two weeks | Hangzhou UEBMI | 33.8% | 26.5% | 39.5% | 43.8% | 43.3% |  |
|  | Hangzhou URBMI | 48.7% | 51.1% | 49.1% | 34.5% | 32.7% |  |
|  | Baoji UEBMI | 50.8% | 33.3% | 32.0% | 35.9% | 46.6% |  |
|  | Baoji URBMI | 50.7% | 44.2% | 37.0% | 30.4% | 47.9% |  |

OOP: out-of-pocket; UEBMI: Urban employee basic medical insurance; URBMI: Urban resident basic medical insurance.
